# Chinese dentists’ restorative preferences and choices for endodontically treated teeth: a representative survey

**DOI:** 10.1186/s12903-024-05217-2

**Published:** 2024-12-18

**Authors:** Wenhui Li, Ziting Zheng, Yuting Zeng, Zhiyan Zhou, Ping Xiao, Xincen Zhong, Wenjuan Yan

**Affiliations:** 1 Department of Stomatology, Nanfang Hospital, Southern Medical University, 1838 N Guangzhou RD, Guangzhou, PR China; 2 Department of Stomatology, Longgang Otorhinolaryngology Hospital, Shenzhen, Guangdong China

**Keywords:** Surveys and questionnaires, Endodontically treated teeth, Dental restoration, Endocrown, Dental practitioners

## Abstract

**Background:**

The optimal restoration protocol for endodontically treated teeth (ETT) remains a subject of debate. This survey aims to assess the current level of awareness, knowledge, and prevailing opinions among dental practitioners in China regarding the application of endocrown versus the post/core/crown ensemble for post-endodontic restoration strategies.

**Methods:**

A validated questionnaire, encompassing three sections, was distributed electronically to dentists practicing in China. The initial section collected demographic characteristics of the participants, while the subsequent sections assessed their knowledge and preferences regarding ETT restoration techniques in various clinical scenarios. Distribution of the survey was facilitated through the social media platform WeChat, with a total of 600 invitations sent out. Data analysis was conducted using SPSS Statistical Software, employing frequency and Chi-square tests to determine statistical significance at the *P* < 0.05 threshold.

**Results:**

A total of 400 valid questionnaires were collected. The amount of remaining tooth structure was identified as the most influential factor in determining the restoration strategies, contributing to 26.1%. Over 72.8% of the surveyed dentists acknowledged the reinforcing effect of intraradicular posts on ETT. More than half of the participants reported the application of endocrowns within their post-endodontics management. The preference for endocrowns was pronounced in cases where more than 50% of the tooth’s structure remained or when occlusal space limitations were present. The Chi-Square test revealed that the participants’ knowledge regarding endocrown restoration was significantly influenced by their age, educational background, and experience (*p* < 0.05).

**Conclusions:**

The clinical decision-making process for the restoration of endodontically treated teeth (ETT) by dental practitioners primarily relies on the amount of remaining tooth structure. Most surveyed dentists believe that the presence of a post can reinforce ETT. A majority of participants consider the Endocrown as a viable alternative restorative treatment for ETT.

## Background

The reconstruction of endodontically treated teeth (ETT) that have undergone extensive destruction of their dental crowns presents a significant challenge in dental practice. The structural changes in dentin and the compromised integrity of ETT lead to decreased fracture resistance and increased risk of biomechanical failure, compared to vital teeth [1, 2]. The primary objective of post-endodontic management aims to achieve minimally invasive tooth preparation while maximizing the conservation of tissues surrounding the restored teeth [3, 4]. Traditionally, restorative strategies for ETT involve direct filling, full-coverage crowns, post-core-crowns, and so on. These approaches have been shown to effectively enhance the resistance of endodontically treated posterior teeth to occlusal forces, contributing to a more favorable long-term prognosis [5]. However, the potential risks associated with post-placement, including root perforation and fracture, must be carefully considered [6]. Indeed, post-placement-related complications have been identified as the second most common cause of tooth structural loss in clinical practice [7].

Endocrowns have emerged as a promising alternative to traditional treatment approaches, eliminating the necessity for post and core build-up. This advancement is attributed to significant improvements in adhesive techniques and the growing emphasis on minimally invasive treatment options [8, 9]. Distinct from conventional approaches, endocrowns are monolithic restorations that are anchored to the pulp chamber and cavity margins through both macro- and micro-mechanical retention, achieved by the pulpal walls and adhesive cementation, respectively [10, 11]. Owing to its core-crown integrated structure, endocrowns offer the advantage of sealing access to the root canal system and minimizing bacterial microleakage. In vitro researches have indicated that the fracture strength of endocrowns is either comparable to or surpasses that of traditional crowns [12–14]. Additionally, it can be fabricated using Computer-aided design and computer-aided manufacturing (CAD/CAM) technology, which enables the fabrication and placement of a complete restoration within a single dental office visit, benefiting both patients and practitioners [15]. Alongside the prevalence of CAD/CAM, various materials continue to advance, aiming to mimic the mechanical and optical properties of natural teeth [16–19]. Dentists can select suitable materials for endocrowns, such as ceramic, hybrid ceramic, or composite resins, tailored to the specific needs of each case.

Having a comprehensive understanding of various dental treatment alternatives is crucial for dentists to effectively address patients’ needs and achieve long-lasting restorative dentistry outcomes. Specifically, post-endodontic restorations require careful consideration of factors such as the structural integrity of the tooth, patients’ aesthetic preferences, and the need to protect the remaining tooth structure [20]. Moreover, dentists’ clinical experience, postgraduate training, and personal opinions play a significant role in influencing the decision-making process [19, 21–24]. Thus, it is crucial to consider dentists’ knowledge and opinions toward treatment options in a clinical context to provide reliable scientific evidence. Understanding their perspectives can provide valuable insights into how treatment decisions are made in practice, help identify potential gaps in knowledge or biases, and ultimately lead to more informed and evidence-based treatment recommendations [25].

Surveys serve as invaluable tools for assessing and gaining insights into the treatment approaches and decision-making processes for restoring ETTs. They facilitate the identification of knowledge gaps and inform educational efforts, thereby ensuring the adoption of evidence-based practices in this specialized area of dentistry [24]. Despite the availability of such data in regions like Saudi Arabia [19, 21, 26], Turkey [23], and Germany [27], the strategies and preferences of Chinese dental practitioners regarding ETT restorations remain unexplored. Consequently, this study aims to evaluate the knowledge and experience of Chinese dentists concerning their approach to restoring ETTs, with a focus on the utilization of endocrowns versus other traditional restorative methods.

## Methods

This survey was conducted among dental practitioners from diverse institutions across China, adhering to ethical guidelines approved by the Medical Ethics Committee of Nanfang Hospital, Southern Medical University (Approval Number: NFEC-2017-141). Data were gathered using a self-administered questionnaire crafted with Questionnaire Star (Changsha Ranxing Information Technology Co., Ltd., Changsha, China), a reputable online survey platform. The questionnaire link was disseminated through the widely used social media platform WeChat (Tencent, Shenzhen, China) to enhance its accessibility among dental professionals nationwide. To uphold the statistical rigor of this study, G*Power Version 3.1.9 was employed to determine the requisite sample size, ultimately concluding that a minimum of 377 respondents would be necessary to attain a study power of 80%. Consequently, a total of 600 invitations were disseminated via WeChat, targeting a diverse group of dental professionals, including endodontists, general practitioners, restorative specialists, and others actively engaged in ETT management.

The questionnaire used in this study was developed by the authors, incorporating insights from published studies and expert opinions to ensure its relevance. Furthermore, the questionnaire underwent evaluation for both reliability and validity [19, 21]. It consisted of three sections. The first section collected demographic data, including gender, age group, education level, clinical title, specialty, years of experience, and workplace. The second section featured 11 questions designed to elucidate participants’ preferences for restorative procedures for ETTs across different clinical scenarios. The final section comprised 5 questions investigating participants’ experiences with the application of endocrowns.

The questionnaire link remained accessible for a comprehensive three-month period, from February 2023 to April 2023. To bolster participation rates, follow-up reminders were dispatched twice, spaced three weeks apart, subsequent to the initial invitation. To uphold data quality standards, submissions completed in less than 30 s or exceeding 30 min were excluded from the analysis, ensuring that only valid responses were taken into consideration. Data analysis was performed using SPSS 16 software (SPSS, Inc., Chicago, IL, USA), employing frequency tests and Chi-square tests with the significance level set at 0.05.

## Results

Out of 415 responses collected, 400 questionnaires were deemed eligible for inclusion in the study, yielding a participation rate of 69.2%. The demographic details of these participants are summarized in Table 1. Among the participants, the largest proportion (54.5%) belonged to the 26–35 age group. In terms of education level, 44.3% of participants had a bachelor’s degree, followed by 30.5% with a master’s degree. Regarding professional experience, 55.5% of participants had less than 6 years of clinical practice, and 49.8% were clinical dentists. The largest specialty group was represented by general dental practitioners (39.5%), closely followed by endodontists (33.3%). Most participants (49.3%) worked primarily in the dentistry department of a general hospital.

**Table 1:** Socio-demographic characteristics of participants in the survey

Characteristics	Options	Number	Percentage
Gender	Male	170	42.5
	Female	230	57.5
Age Group	< 26	101	25.3
	26–35	218	54.5
	36–45	58	14.5
	> 45	23	5.7
Education Level	College Degree	75	18.7
	Bachelor’s Degree	177	44.3
	Master’s Degree	122	30.5
	Doctoral Degree	26	6.5
Clinical Title	Assistant Dentist	89	22.3
	Dentist	199	49.8
	Dentist in Charge	87	21.8
	Associate Chief Dentist	15	3.8
	Chief Dentist	10	2.5
Specialty	Restorative Dentistry	61	15.3
	Endodontists	133	33.3
	General Dental Practitioners	158	39.5
	Other	48	12.0
Years of Experience	< 6	222	55.5
	6–10	93	23.3
	> 10	85	21.3
Workplace	Specialist Hospital	118	29.5
	General Hospital	197	49.3
	Private Clinic	73	18.3
	Other	12	3.0

**Fig. 1:**
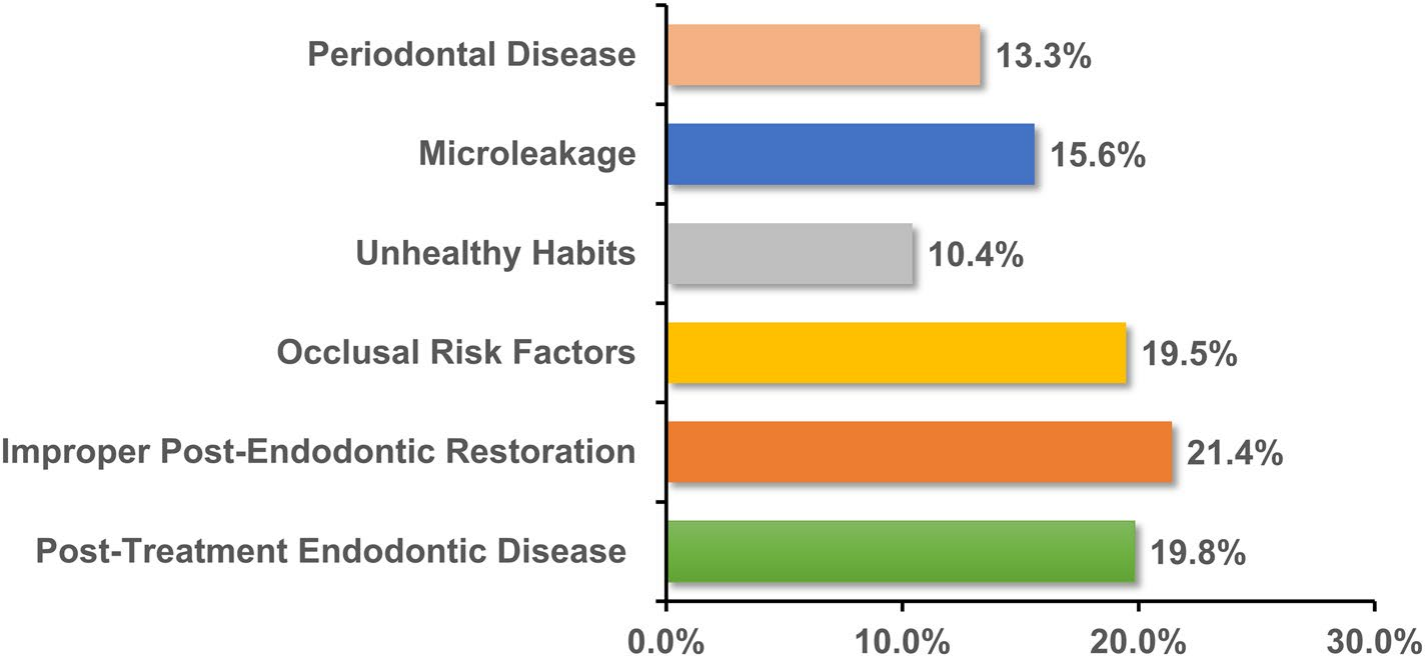
Dentists’ perceptions of reasons for failure of ETT

**Fig. 2:**
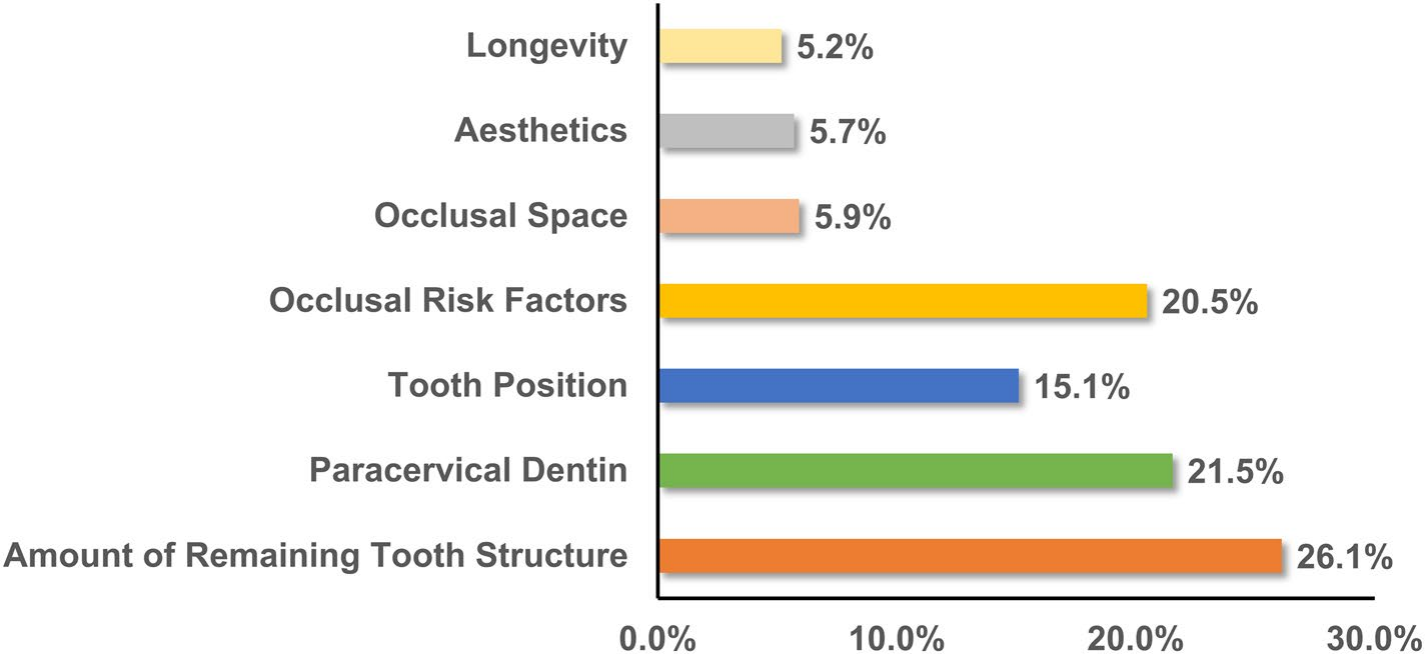
Primary consideration for choosing the restoration of ETT

Regarding dentists’ perceptions of the reasons for the failure of ETT, three primary factors were identified: improper post-endodontic restorations (21.4%), post-treatment endodontic disease (19.8%), and occlusal risk factors (19.5%), as illustrated in Fig. 1. When planning restorations for ETT, dentists prioritized the remaining tooth structure (26.1%), followed closely by the paracervical dentin (21.5%) and occlusal risk factors (20.5%), as shown in Fig. 2. The assessment of clinical restoration options for tooth structure loss revealed that for ETT with four axial walls, crowns were the preferred choice (40.8%), followed by endocrowns (26.5%) and direct fillings (23.0%). However, for ETT with one or two residual walls, most dentists favored the post-and-core system, with preference ranging from 53.3 to 61.8%, while the preference for endocrowns ranged from 14.5 to 20.5% (Table 2).

**Table 2:** Preferred restorative treatment according to the amount of residual axial walls

Restoration	Four axial walls		Three axial walls		Two axial walls		One axial wall	
	*N*	%	*N*	%	*N*	%	*N*	%
Direct Filling	92	23.0	24	6.0	12	3.0	22	5.5
Endocrown	106	26.5	135	33.8	82	20.5	58	14.5
Singe Crown	163	40.8	150	37.5	93	23.3	73	18.4
Post-and-Core	39	9.8	91	22.8	213	53.3	247	61.8

Table 3 presents participant responses regarding the reinforcing effect of intraradicular posts on ETT, revealing statistically significant differences (*p* < 0.001). 72.8% of participants believed that ETT would be more resistant after post-and-core restoration, while 27.2% disagreed. Preferences for post-reinforcement of ETT were significantly associated with demographic characteristics, including age, education level, clinical title, and years of experience (*p* < 0.05). Younger participants (ages 26 to 35) were more likely to believe that posts could increase ETT resistance. Specifically, 82.7% of participants with an associate degree supported this view compared to 50% of those with doctoral degrees. Regarding different clinical titles, assistant dentists had the highest percentage of belief in post-reinforcement (85.4%). Participants with less than 6 years of experience were more likely to support the idea that post-reinforcement could increase the resistance of ETT.

**Table 3:** Analysis of responses to the belief that intraradicular posts can reinforce ETT, based on age, education level, clinical title, and years of experience

Characteristics	Options	Yes			No	χ²	*P
		*N*(%)		*N*(%)			
Age Group	< 26	88(87.1)		13(12.9)		17.4	< 0.001
	26–35	154(70.6)		64(29.4)			
	36–45	36(62.1)		22(37.9)			
	> 45	13(56.5)		10(43.5)			
Education Level	College Degree	62(82.7)		13(17.3)		12.4	< 0.05
	Bachelor’s Degree	133(75.1)		44(24.9)			
	Master’s Degree	83(68.0)		39(31.9)			
	Doctoral Degree	13(50.0)		13(50.0)			
Clinical Title	Assistant Dentist	76(85.4)		13(14.6)		26.2	< 0.000
	Dentist	147(73.9)		52(26.1)			
	Dentist in Charge	52(59.8)		35(40.2)			
	Associate Chief Dentist	12(80.0)		3(20.0)			
	Chief Dentist	4(40.0)		6(60.0)			
Years of Experience	< 6	174(78.4)		48(21.6)		10.0	< 0.05
	6–10	61(65.6)		32(34.4)			
	> 10	56(65.9)		29(34.1)			
Total		291(72.8)		109(27.2)			

**Fig. 3:**
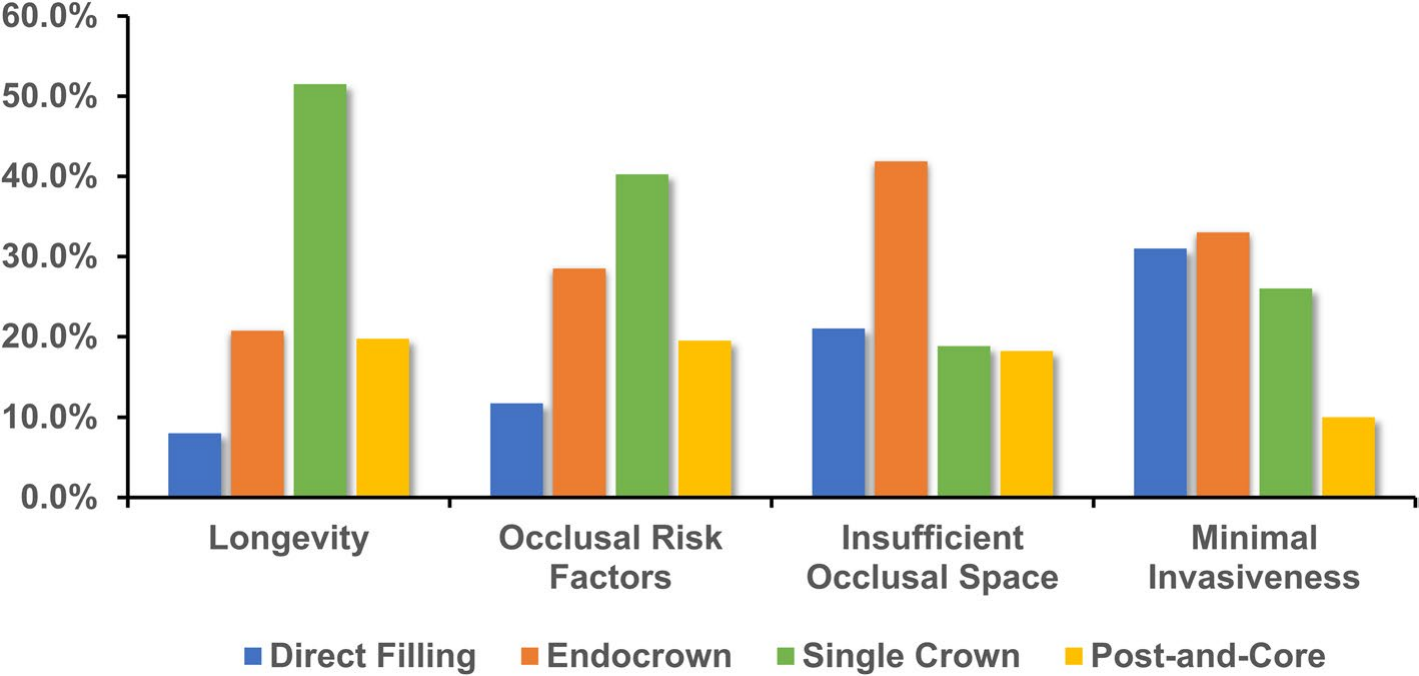
Preferred restorative options for ETT based on varying clinical objectives

When prioritizing the longevity of ETT, a significant majority of participants (51.5%) favored the single crown as the preferred restoration method (Fig. 3). In scenarios where patients presented with occlusal risk factors, 40.3% of respondents preferred employing a single crown for restoration. For patients with insufficient occlusal space, 41.9% of participants considered the endocrown as the most preferred restoration option. Regarding minimally invasive repair, 33.0% of participants identified the endocrown as their top choice for restoring ETT.

In the application of endocrown restorations, 56.3% of respondents reported using this technique, significantly influenced by demographic factors, as detailed in Table 4. The age group 36 to 45 years showed the highest utilization rate of 81.0%. High-level clinicians, such as associate chief dentists (86.7%), chief dentists (80.0%), and dentists in charge (79.3%), preferred endocrown restorations. General dental practitioners had a utilization rate of 61.4%, outperforming their peers in other specialties. A positive association was observed between years of experience and the adoption of endocrown restorations (χ²=67.6, *p* < 0.05), with practitioners having over 10 years (76.5%) and 6–10 years (80.7%) of experience demonstrating higher adoption rates compared to those with less than 6 years (38.3%). When assessing the most common tooth position for endocrown application, 88.4% of respondents used it in the molar region, while 6.7% and 4.9% respectively applied it in the anterior tooth and canine tooth. In assessing the preferences for marginal form in endocrown, it was revealed that 59.6% of the participants favored the 90-degree shoulder as the most commonly used form (Table 5). Furthermore, 65.0% of participants selected ceramic as the most frequently used material for endocrown restorations.

**Table 4:** Prevalence of using endocrowns for managing ETT based on gender, age, clinical title, specialty, and years of experience

Characteristics	Options	Yes		No	*χ²*	*P
		N(%)		N(%)		
Gender	Male	112(65.9)		58(34.1)	11.2	0.001
	Female	113(49.1)		117(50.9)		
Age Group	< 26	35(34.7)		66(65.4)	38.3	< 0.001
	26–35	125(57.3)		93(42.7)		
	36–45	47(81.0)		11(19.0)		
	> 45	18(78.3)		5(21.7)		
Clinical Title	Assistant Dentists	33(37.1)		56(62.9)	43.2	< 0.001
	Dentist	102(51.3)		97(48.7)		
	Dentist in Charge	69(79.3)		18(20.7)		
	Associate Chief Dentist	13(86.7)		2(13.3)		
	Chief Dentist	8(80.0)		2(20.0)		
Specialty	Restorative Dentistry	37(60.7)		24(39.3)	7.8	< 0.05
	Endodontists	72(54.1)		61(45.9)		
	General Dental Practitioners	97(61.4)		61(38.6)		
	Other	19(39.6)		29(60.4)		
Years of Experience	< 6	85(38.3)		137(61.7)	67.6	< 0.001
	6–10	75(80.7)		18(19.4)		
	> 10	65(76.5)		20(23.5)		
Total		225(56.3)		175(43.8)		

**Table 5:** Practitioners’ preference for the clinical application of endocrown restorations

Questionnaire		*N*	(%)
Which tooth position do you commonly apply for endocrown?			
a	Anterior teeth	15	6.7
b	Canine	11	4.9
c	Molars	199	88.4
Which marginal form do you commonly use for endocrown?			
a	Flat butt joint	91	40.4
b	90-degree shoulder	134	59.6
What material do you commonly use for endocrown?			
a	Ceramic	147	65.0
b	Hybrid ceramic	44	19.7
c	Composite resin	34	15.3

Upon analyzing the responses to the questions regarding whether endocrown could serve as an alternative restorative treatment for managing ETT, it was observed that over half of the participants (63.8%) shared this view (Table 6). Three demographic characteristics (education level, specialty, and workplace) exhibited significant differences in attitudes towards the endocrown as an alternative treatment. Specifically, those with advanced education levels, particularly those specializing in endodontics and working in general hospitals, exhibited a greater tendency to endorse the potential of endocrown as an alternative restorative treatment.

**Table 6:** Prevalence of using endocrowns as an alternative treatment option for managing ETT based on education level, specialty, and workplace

Characteristics	Options	Yes	No	Uncertain	χ²	*P
		*N*(%)	*N*(%)	*N*(%)		
Education Level	College Degree	41(54.7)	7(9.3)	27(36.0)	14.3	0.027
	Bachelor’s Degree	117(66.1)	12(6.8)	48(27.1)		
	Master’s Degree	75(61.5)	18(14.8)	29(23.8)		
	Doctoral Degree	22(84.6)	2(7.7)	2(7.7)		
Specialty	Restorative Dentistry	35(57.4)	8(13.1)	18(29.5)	18.6	0.005
	Endodontics	98(73.7)	13(9.8)	22(16.5)		
	General Dental practitioners	101(63.9)	13(8.2)	44(27.9)		
	Others	21(43.8)	5(10.4)	22(45.8)		
Workplace	Specialist Hospital	66(55.9)	15(12.7)	37(31.4)	14.1	0.029
	General Hospital	139(70.6)	16(8.1)	42(21.3)		
	Private Clinic	45(61.6)	8(11.0)	20(27.4)		
	Other	5(41.7)	0(0.0)	7(58.3)		
Total		255(63.8)	39(9.8)	106(26.5)		

## Discussion

This survey was conducted to investigate the treatment strategies and preferences used in ETT restoration in China. We successfully collected 400 valid questionnaires, achieving a participation rate of 69.2%, which aligns with the methodology employed in similar studies [26]. This high participation rate underscores the engagement and interest of practitioners in the topic under investigation, thereby enhancing the reliability and representativeness of the findings.

The long-term success of ETT is influenced by factors such as post-treatment endodontic disease, improper post-endodontic restoration techniques, as well as occlusal risks, and other potential complications [28]. Our survey identified improper post-endodontic restoration as the primary cause of ETT failure (21.4%), as shown in Fig. 1. Prior research shows that combining root canal treatments with adequate restoration elevates the success rate to 91.4%, whereas inadequate restoration significantly reduces it to 44.0% [29]. This underscores the importance of proper restoration in preventing micro-leakage and mitigating long-term endodontic failures [30]. In selecting restoration options for ETT (Fig. 2), participants prioritized the remaining tooth structure volume as a crucial determinant. This perspective is validated by research [31], which indicates a 2.58-fold increased risk of unfavorable outcomes in teeth retaining < 30% of their original volume at one-year follow-up compared to those with > 30% volume. This supports the principle of preserving tooth tissue, achieved through minimally invasive techniques.

Dental practitioners employ diverse treatment planning strategies when evaluating restorations for tooth structure loss. A literature review advocates ETT restoration for limited tissue loss, specifically when over 50% of coronal structure remains, without post-placement, particularly for cusp protection. In such scenarios, complete occlusal coverage techniques are recommended [32]. Consistent with this, our survey shows single crowns as the primary restoration method for ETTs with > 50% coronal structure, followed by endocrowns (Table 2). Moreover, with the rise in minimally invasive restoration techniques, the literature favors medium-term outcomes for direct fillings in root-filled teeth, aligning with our findings where 23.0% of participants opted for this method. In cases of ETT with over 50.0% coronal structure loss, mounting evidence favors the use of endocrowns. Rasidi and Priscilla’s study reports that 42.5% of dental practitioners prefer endocrowns for teeth lacking three walls and 35.0% for those missing two walls [33]. This preference is echoed in a survey conducted in Ha’il, where 37.3% of participants endorse endocrowns for crowns with more than half of their structure damaged [21]. However, a notable discrepancy exists in China (Table 2), where post-core crown restoration is predominantly favored (53.4%), with endocrowns enjoying relatively low adoption rates (< 20.5%). This variation underscores the importance of raising awareness and promoting the use of endocrowns, particularly in cases involving severe proximal wall damage.

As shown in Table 3, a majority of respondents (72.8%) in this survey believed in the reinforcement effect of an endodontic post, a view shared by 42.9% of practitioners in Germany and 58% in Saudi Arabia [19]. However, current evidence-based knowledge contradicts this belief, indicating that posts do not reinforce the root or tooth but rather support the core build-up. Notably, the belief in post-reinforcement was significantly associated with factors such as age, education level, clinical title, and years of experience (*p* < 0.05). Younger participants (< 36 years) with less clinical experience and lower educational backgrounds were more likely to hold this outdated belief, potentially influenced by dental school teachings and obsolete literature. To address this, there is a pressing need for further implementation of continuing education and development courses in China, aimed at updating practitioners’ knowledge and practices in line with current evidence-based standards.

Endocrowns are particularly advantageous in situations where traditional post-and-core systems are challenging to apply. They are effective alternatives for teeth with short clinical crowns, curved or calcified root canals, or when fractured instruments obstruct post-placement [8]. By leveraging adhesive bonding within the pulp chamber, endocrowns minimize reliance on residual tooth structure. However, certain contraindications must be considered. A shallow pulp chamber may not provide sufficient bonding surface, leading to instability [34]. Additionally, parafunctional habits and specific occlusal anatomical issues, such as excessive lateral stress from steep occlusal surfaces, wear, or facets, can adversely affect the durability of endocrowns.

Upon inquiry regarding the clinical application of endocrowns, a substantial proportion of dental practitioners concur that endocrowns are primarily indicated for molars, with limited application to anterior teeth and canines. This aligns with a prior Riyadh-based research emphasizing molars as the preferred site [33]. Although Sevimli et al. suggested endocrowns as a prosthetic alternative for ETT, including incisors, premolars, and molars, further research indicates that their performance is optimized in posterior teeth [35]. This is attributed to the larger pulp chamber and axial loading under functional stress in premolars and molars. A deeper analysis of the survival rates of endocrowns in premolars and molars reveals a significantly higher failure rate in premolars compared to molars. This disparity is attributed to the smaller pulp chamber, which restricts the adhesive bond strength, and the unique crown shape increases fracture risks [36]. Consequently, endocrowns are advocated for molars, especially those with shorter crowns, calcified pulp chambers, or narrow root canals [19, 37].

In designing endocrown preparations, two common margin forms are the 90-degree shoulder and the flat butt joint [38]. The flat butt margin preserves tissue, offers superior clinical maneuverability, and has low technical sensitivity; while the 90-degree shoulder margin mitigates shear stresses, enhances load distribution, increases adhesive surface area, and minimizes bonding failure risks [39, 40]. Our study revealed a preference for the 90-degree shoulder among 59.6% of dentists, echoing a Chennai survey where a majority emphasized the importance of a ferrule in endocrown preparations [39]. Interestingly, demographic analysis disclosed differences in margin preferences among specialists. Prosthodontists (73.0%) and general practitioners (61.9%) leaned towards the 90-degree shoulder, whereas endodontists (54.2%) favored the flat butt margin. This divergence underscores the significance of considering practitioner expertise and individual treatment approaches when selecting the most suitable margin form for endocrown preparations.

When questioned about commonly used materials for endocrowns, dental practitioners overwhelmingly prefer ceramics due to their aesthetics, biocompatibility, and mechanical strength. This aligns with prior Saudi Arabia and Jordan-based studies, which identified lithium disilicate as the preferred material [33, 41]. However, ceramics are prone to brittle catastrophic fracture and excessive wear on opposing teeth, particularly in patients with tight occlusal spaces, poor occlusal habits, or limited occlusal space. In such cases, hybrid ceramics and composite resins, which exhibit lower hardness and a similar elastic modulus to dentin, can be viable alternatives [42]. Therefore, dentists should consider a patient’s specific needs and occlusal condition when selecting the appropriate material for endocrown restorations.

In this study, when assessing the application of endocrowns and evaluating their feasibility as an alternative treatment modality, a demographic Chi-square analysis revealed that older, experienced clinicians with doctoral degrees and high professional titles demonstrated a propensity towards approval. This association underscores the importance of continuing education and clinical experience in the adoption of endocrowns. Notably, the current Chinese dental curricula for undergraduates and junior colleges lack comprehensive coverage on pulp cavity retainer crowns, potentially impacting their acceptance among practitioners. This highlights the necessity to integrate advanced techniques and materials into dental education to enhance future practitioners’ clinical proficiency.

Several limitations should be considered in interpreting this study. First, the limited sample size may not fully capture the characteristics of the broader population, and the exclusive focus on Chinese dentists constrains the global relevance of the findings. Although the cross-sectional approach provides a snapshot of current practices, it lacks the capacity to observe trends over time or assess the impact of educational interventions. Additionally, the study relies on self-reported survey data, which is prone to bias since practitioners may report ideal practices rather than actual behaviors, potentially impacting the reliability of findings regarding the knowledge-to-practice gap. The sample shows a preponderance of younger and less experienced general practitioners, thus limiting the generalizability to more experienced or specialized practitioners. Furthermore, the focus on a standardized clinical scenario restricts applicability to the diverse, complex cases encountered in real-world practice. Future research with observational or longitudinal methods, expanded sample diversity, and international collaboration to enhance robustness and relevance.

## Conclusions

Within the limitations of this survey, the following conclusions can be drawn:

1. The clinical decision-making process for the restoration of endodontically treated teeth (ETT) by dental practitioners primarily relies on the amount of remaining tooth structure.
2. Most surveyed dentists believe that the presence of a post can reinforce ETT.
3. A majority of participants consider the Endocrown as a viable alternative restorative treatment for ETT.

## Data Availability

The data can be provided by the corresponding author upon reasonable request.

## Abbreviations

ETT: Endodontically treated teeth
CAD/CAM: Computer-aided design and computer-aided manufacturing
